# A Multidisciplinary Approach of an Endo-Perio Lesion in a Severely Compromised Tooth: An 18-Year Follow-up Case Report

**DOI:** 10.25122/jml-2020-0017

**Published:** 2020

**Authors:** Ramón Pereira, Silie Arboleda

**Affiliations:** 1. Periodontics, Private Practice, Bogotá, Colombia; 2. Universidad Nacional de Colombia, School of Dentistry, Bogotá, Colombia; 3. Unit of Clinical Oral Epidemiology Investigations (UNIECLO), School of Dentistry, Universidad El Bosque. Bogotá, Colombia

**Keywords:** Endo-periodontal lesion, gingival recession, root canal obturation, mucogingival therapy, case report

## Abstract

This case report describes the diagnosis, multidisciplinary treatment, and long-term follow-up of a severely compromised tooth in a patient who was referred for assessing a gingival recession. Clinical evaluation of the left maxillary canine showed 12 mm of mid-buccal gingival recession, probing depth of 14 mm on the mesial-buccal aspect, and grade III mobility. A periapical radiograph revealed extensive periapical and lateral radiolucency. The first step of the treatment was to carry out oral hygiene instructions and full-mouth debridement. After that, endodontic treatment was performed immediately. Periodontal reevaluation four months after endodontic therapy revealed that probing depths of all sites were within 3 mm and periapical radiograph showed a slight decrease in periapical and lateral radiolucency. It was subsequently decided to perform root coverage with a laterally positioned flap and subepithelial connective tissue graft. Six months after surgery, the root surface showed 1 mm recession, representing root coverage of 91.7% and a gain of attachment of 13 mm. The patient was enrolled in a 6-month supportive periodontal therapy. Treatment outcomes were evaluated over 18 years, with successful radiographic and clinical results throughout the follow-up period. The successful management of endo-periodontal lesions requires an accurate diagnosis, which is imperative to provide proper therapy in the correct treatment sequence.

## Introduction

Gingival recession (GR) is the exposure of the root surface due to an apical shift of the gingival margin [1]. The etiology is multifactorial, and it is related to anatomical, physiological, and pathological factors [2]. Orthodontic movement is one of the physiological factors most often associated with GR [3]; specifically, the movement of teeth to positions outside the labial or lingual alveolar plate could lead to dehiscence formation that may subsequently develop GR [4]. Likewise, the orthodontic movement could jeopardize the dental pulp [5]. Published histological studies have reported that the pulpal response to orthodontic force involves cell damage, inflammation, and wound healing, which are processes that could adversely affect the dental pulp [5].

Since the dental pulp and the periodontium are related through different channels of communication, an endodontic lesion that affects the pulp may secondarily affect the periodontium [6]. A periodontal lesion may provoke a pulp pathology [7]. Additionally, both diseases can develop independently [7]. The clinical conditions involving both the pulp and periodontal tissues are called endo-periodontal lesions (EPL) [7]. The most common signs and symptoms associated with EPL are deep periodontal pockets reaching or close to the apex and negative or altered response to pulp vitality tests [6]. Other cases may include tooth mobility, fistula, bleeding on probing, suppuration, and pain [6]. EPL are a clinical dilemma because a precise diagnosis is sometimes difficult. Accurate identification of the etiologic factors is essential for a correct treatment sequence. The prognosis of these lesions depends on the structures involved [6].

This study aimed to present the diagnosis, multidisciplinary treatment, and long-term follow-up of a patient with a severely compromised tooth. It also emphasizes the importance of the correct treatment sequence.

## Case report

A 49-year-old woman patient presented a significant dissatisfaction with the aesthetics due to the gingival recession of a tooth located in the anterior region of the mouth. Dental history revealed that the patient had received orthodontic treatment seven years before. During and after the treatment, she perceived her longer canine tooth.

Clinical examination of tooth 23 revealed the presence of a 12 mm deep and 6 mm wide GR and keratinized tissue of 1 mm width on the buccal surface (Figure 1A). Probing depth (PD), GR and clinical attachment level (CAL) were recorded on six aspects of the tooth (Table 1). Bleeding on probing, slight supragingival calculus deposits, grade III mobility, and GR Class III, according to Miller [8], were also recorded. The initial radiographic image revealed extensive periapical and lateral radiolucency (Figure 1B).

**Table 1:** Preoperative and Postoperative PD, GR and CAL Measurements (mm).

Tooth 23	Baseline						Follow-up of buccal aspect					
	Buccal			Palatal			6 months after surgery			2 years after surgery		
	**M**	B	D	M	P	D	M	B	D	M	B	D
**PD**	13	4	7	3	2	3	3	2	3	3	2	3
**GR**	1	12	0	1	1	1	1.5	1	1	2	2.5	2
**CAL**	14	16	7	4	3	4	4.5	3	4	5	4.5	5

PD: probing depth; CAL: clinical attachment level; GR: gingival recession; Mm: millimeters; M = mesial; B = mid-buccal; D = distal; P = mid-palatal.

**Figure 1A:**
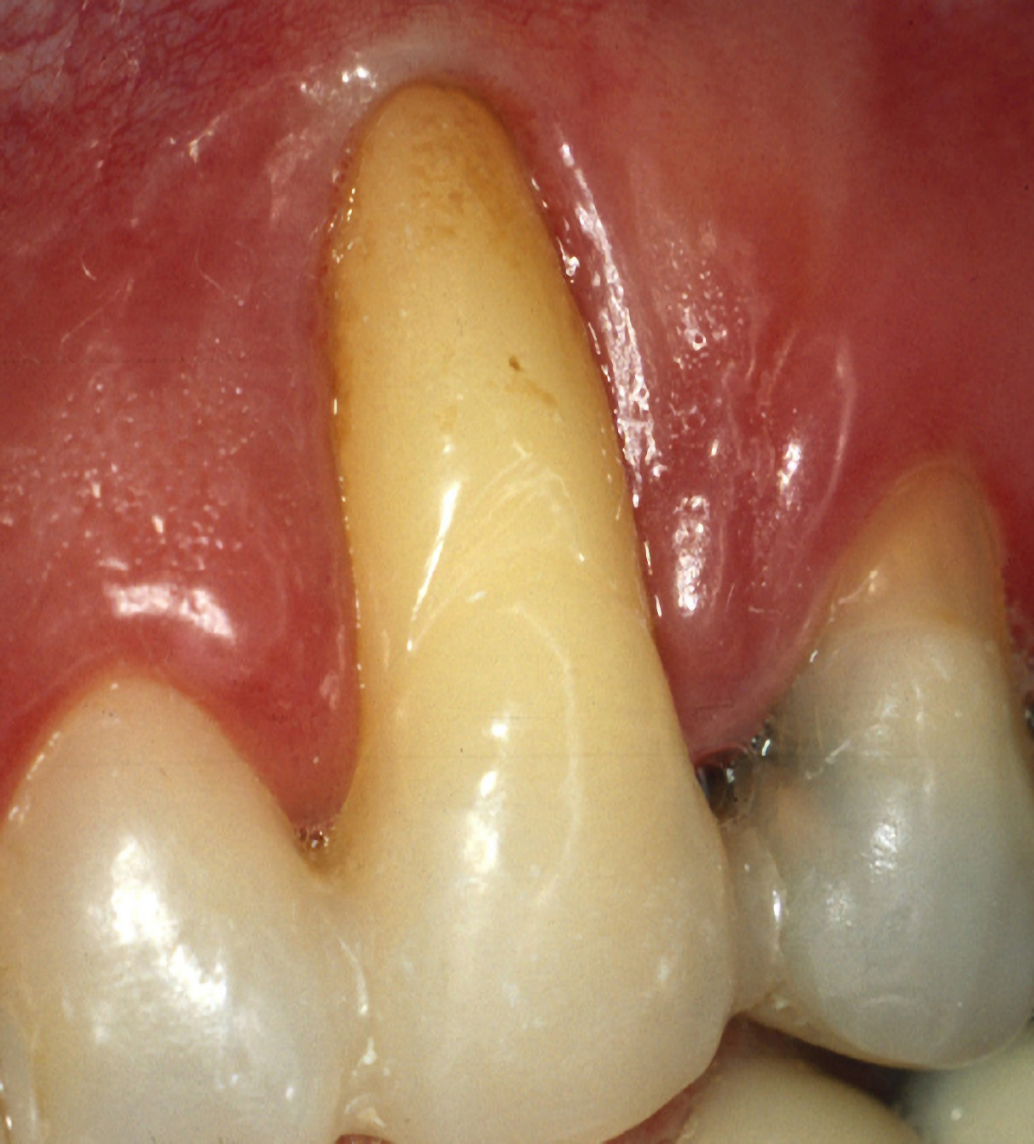
Clinical presentation.

**Figure 1B:**
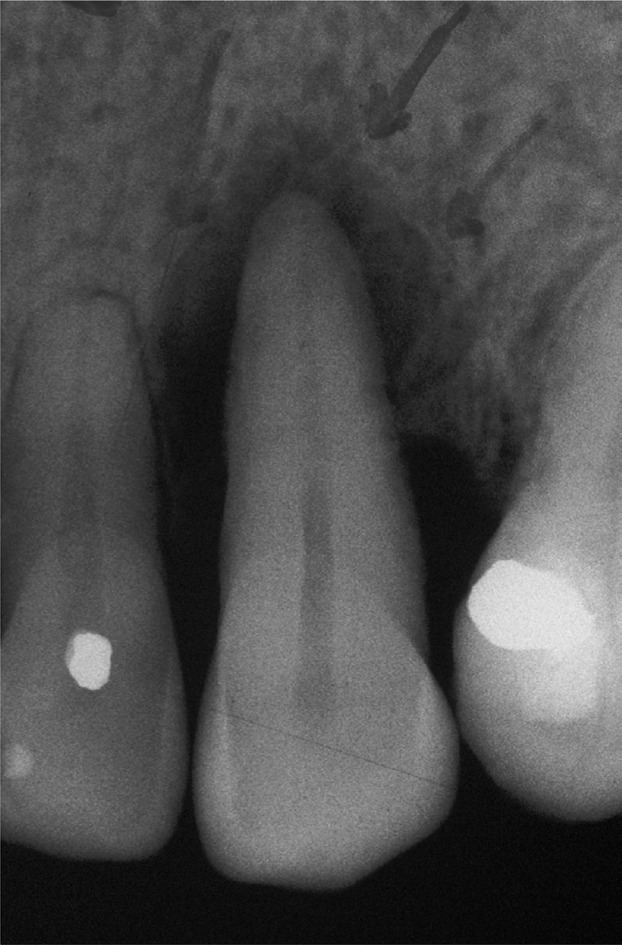
Extensive periapical and lateral radiolucency.

The patient was referred to an endodontist for evaluation. The tooth responded negatively to the cold test, and endodontic treatment was indicated. The tooth was diagnosed as a primary endodontic lesion with secondary periodontal involvement. The patient was informed that the tooth had a poor to guarded prognosis due to severe periodontal attachment loss. Following a discussion of the benefits, risks, and alternative treatment options, she decided to keep her tooth. She approved dental treatment through written informed consent.

**Figure 2:**
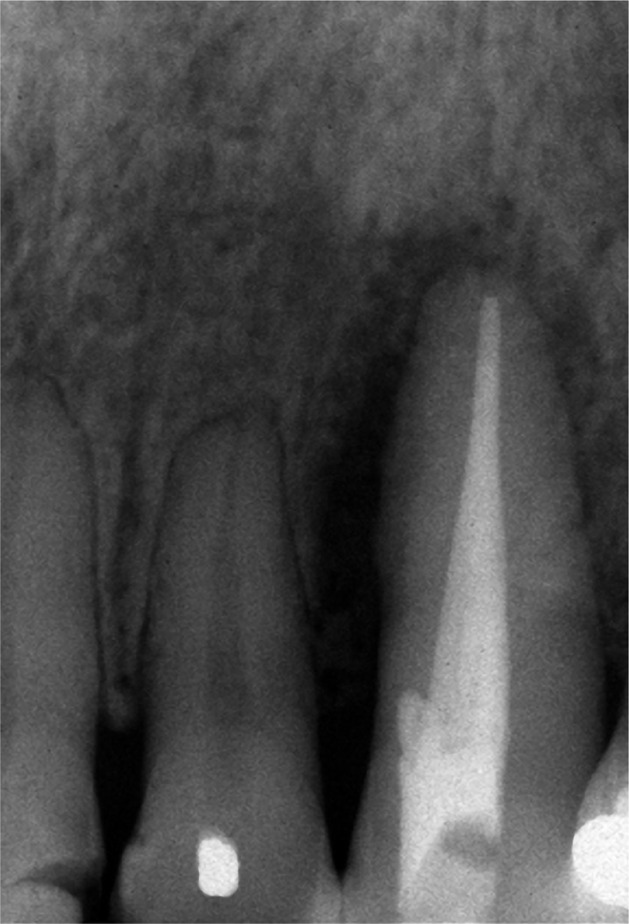
Four months after the endodontic treatment, the radiograph shows a slight decrease in lateral and periapical radiolucency.

The first phase of the treatment consisted of oral hygiene instructions and generalized scaling and root planing. A temporary splint was made to control tooth mobility and improves the patient’s comfort and function. After that, root canal therapy was performed. Four months later, the PD of all sites was within 3 mm, and the periapical radiograph showed a slight decrease in lateral and periapical radiolucency (Figure 2). At that time, it was decided to perform root coverage with a laterally positioned flap (LPF) with subepithelial connective tissue graft (SCTG) on tooth 23. After local anesthesia was provided, an intrasulcular incision was made on the buccal aspect of tooth 23, and a submarginal incision was performed 1.5 mm apical to the gingival margin of tooth 22 with a 15 surgical blade. (Figure 3A). A vertical releasing incision was made extending beyond the mucogingival junction so that the pedicle flap could be reflected without tension. A split-thickness flap was elevated, leaving the periosteum attached to the bone surface. In the region of the mesial and distal papilla of the treated tooth, the epithelium was removed, leaving the vascular connective tissue in place. The exposed root surface was accurately debrided through a sharp curette. The surgical area was dressed in a wet gauze pack, and an SCTG was harvested from the palate. The size of the graft was based on the area of the receptor site. The graft was positioned at the height of the cementoenamel junction and was fixed with 5-0 silk by single and interrupted sutures (Figure 3B). Subsequently, the flap was laterally rotated to cover the exposed tissue and sutured with 5-0 silk. Single interrupted sutures were used to primarily stabilize the flap, provided that no tension was created (Figure 3C). The patient was prescribed 600 mg of ibuprofen and was instructed to rinse with 0.12% chlorhexidine gluconate. Postoperative instructions were to apply ice packs, consume soft food, and avoid brushing the surgical site. The sutures were removed after two weeks. The patient was recalled for professional tooth cleaning every four months for the first year. Afterward, she was enrolled in a 6-month supportive periodontal therapy.

**Figure 3A:**
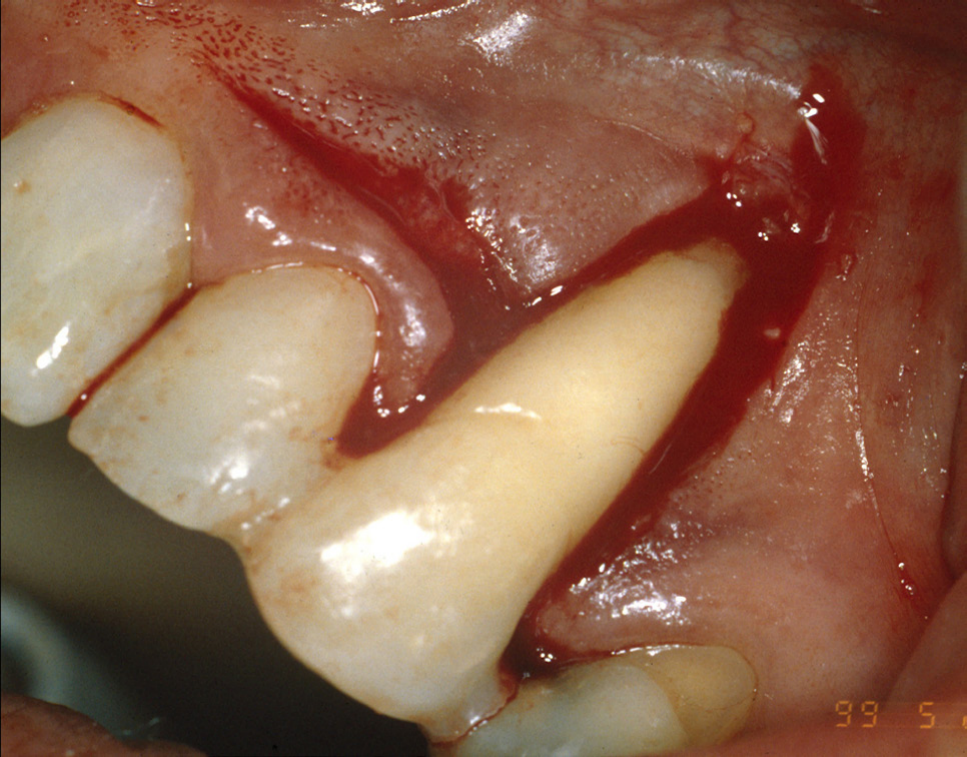
Intrasulcular incision on tooth 23 and submarginal incision on 22.

**Figure 3B:**
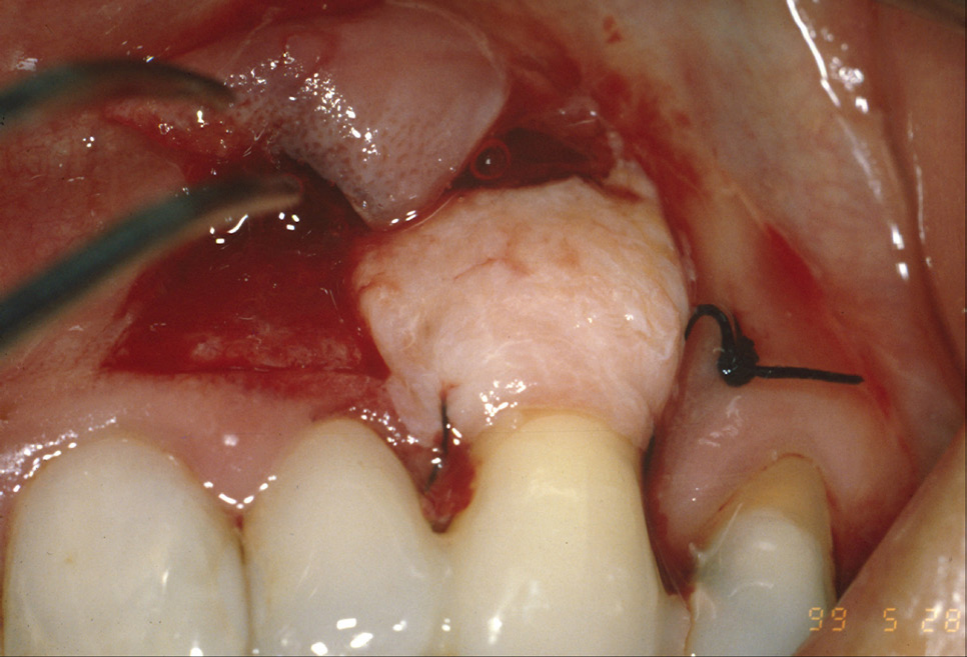
The graft positioned and fixed with 5-0 silk.

**Figure 3C:**
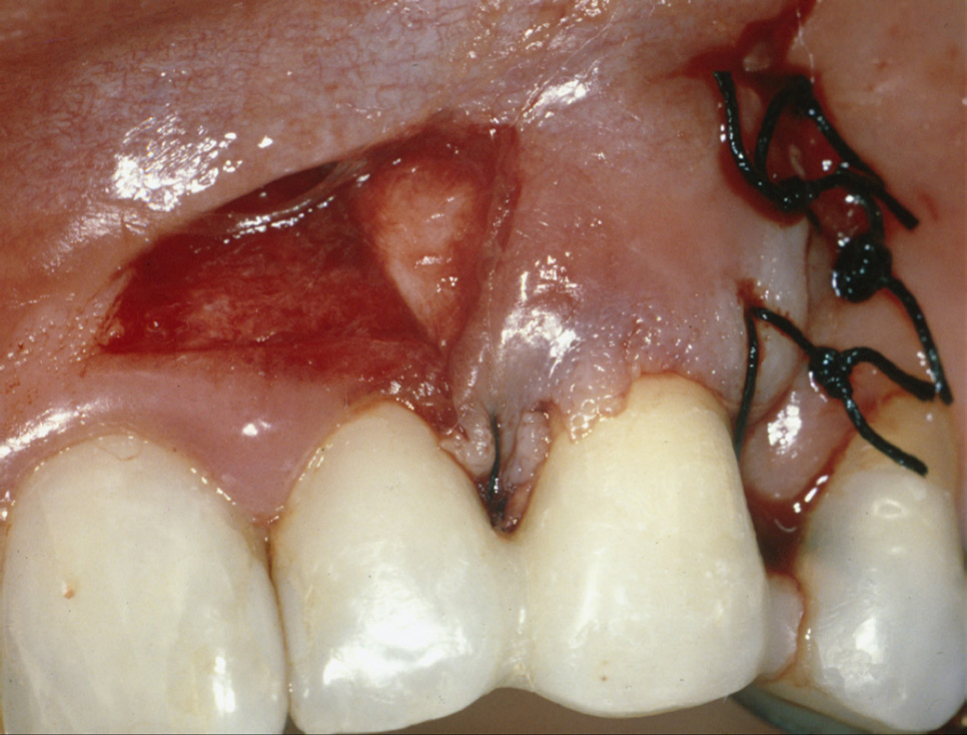
The flap was laterally rotated and sutured.

Postoperative healing was uneventful. At a 6-month follow-up, the canine’s root surface showed 1 mm recession representing root coverage of 91.7% and a gain of attachment of 13 mm, increased buccal keratinized tissue height, and thickness. The root surface of the lateral incisor showed a 1.5 mm recession. (Figure 4A). The radiograph showed a moderate decrease in lateral and periapical radiolucency (Figure 4B).

**Figure 4A:**
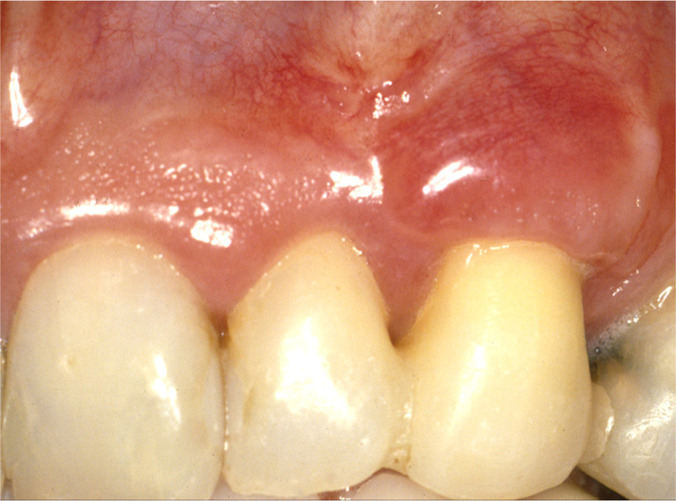
The root surface showed a 1.5- and 1-mm recession on teeth 22 and 23 respectively at 6 months post-surgery.

**Figure 4B:**
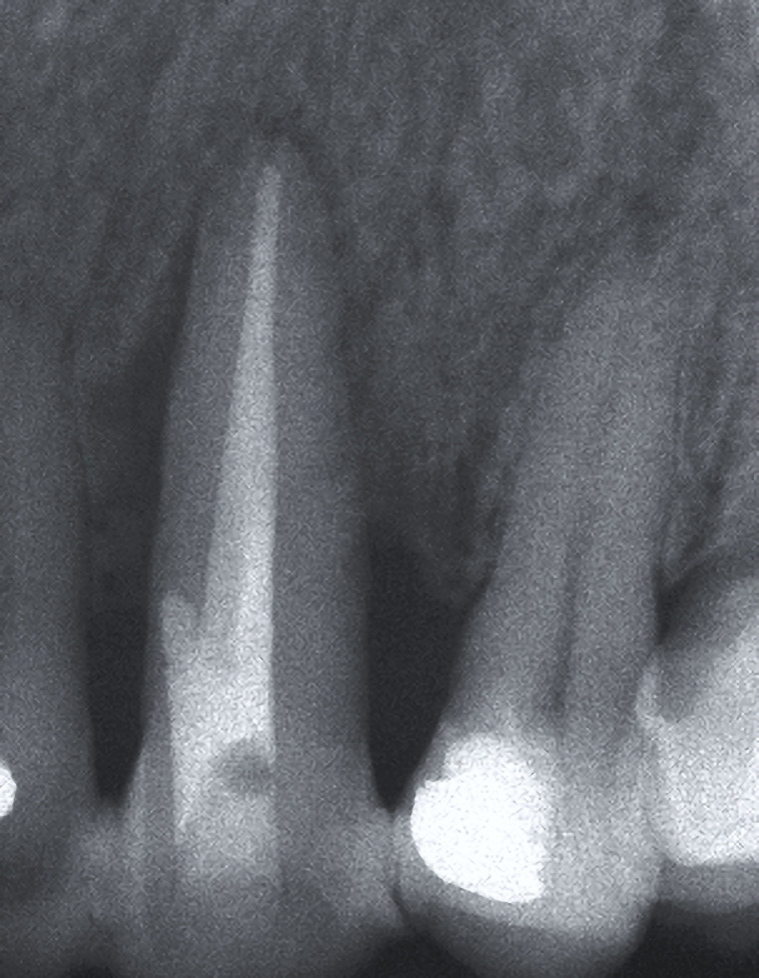
Radiograph showing a moderate decrease in periapical and lateral radiolucency after 6 months.

The patient was very satisfied with the aesthetic appearance of the treated area. The provisional splint was removed, and tooth mobility was almost completely solved (less than grade I). It was decided to perform a buccal and palatal composite splinting to control secondary trauma effects from occlusion. During the periodontal maintenance appointments, probing of the treated area was repeated every six months, while radiographs were taken every year.

Clinical examination at 2- (Figure 5A) and 18-year (Figure 6A) intervals after surgery revealed no increase in PD (3mm), and the root surface showed 2.5 of GR. Radiographic examination at two years showed a slight lateral radiolucency (Figure 5B), and at 18 years, showed complete resolution of the lesion (Figure 6B). During this time, the patient received a crown on tooth 24, which improved the aesthetic result in the treated area.

**Figure 5A:**
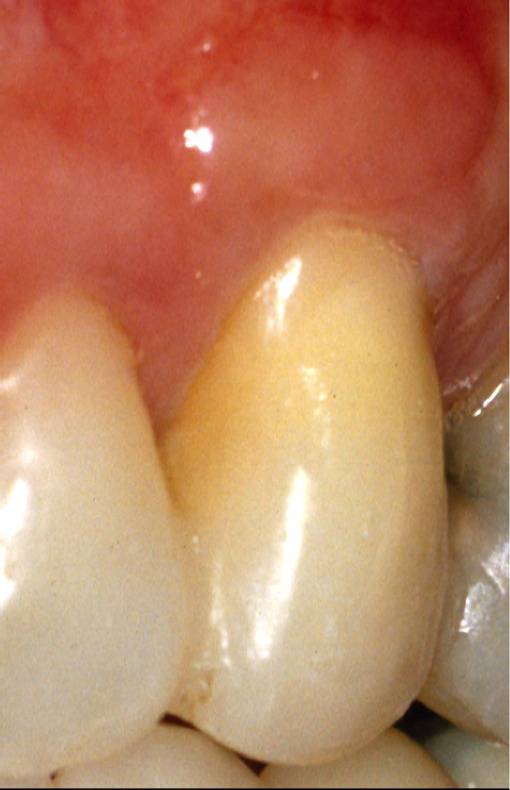
Clinical view with 2.5 mm of GR after 2 years.

**Figure 5B:**
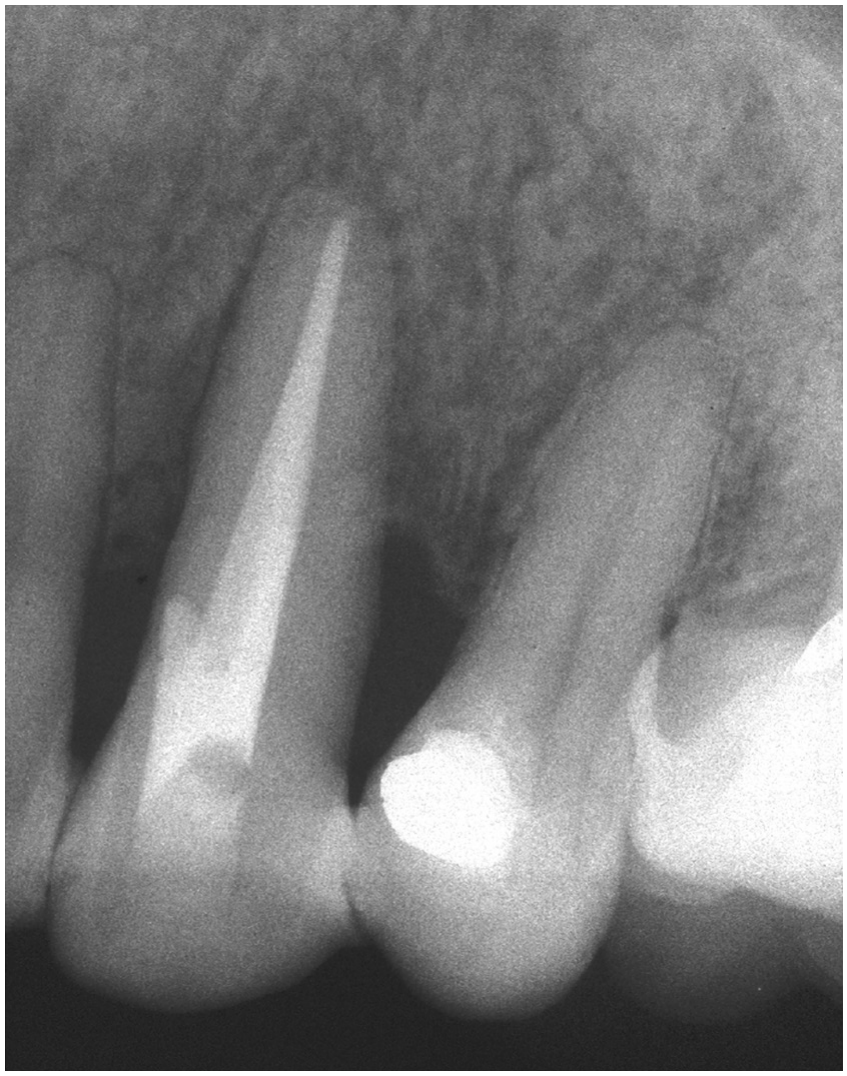
Radiograph shows a slight lateral radiolucency at 2 years follow-up.

**Figure 6A:**
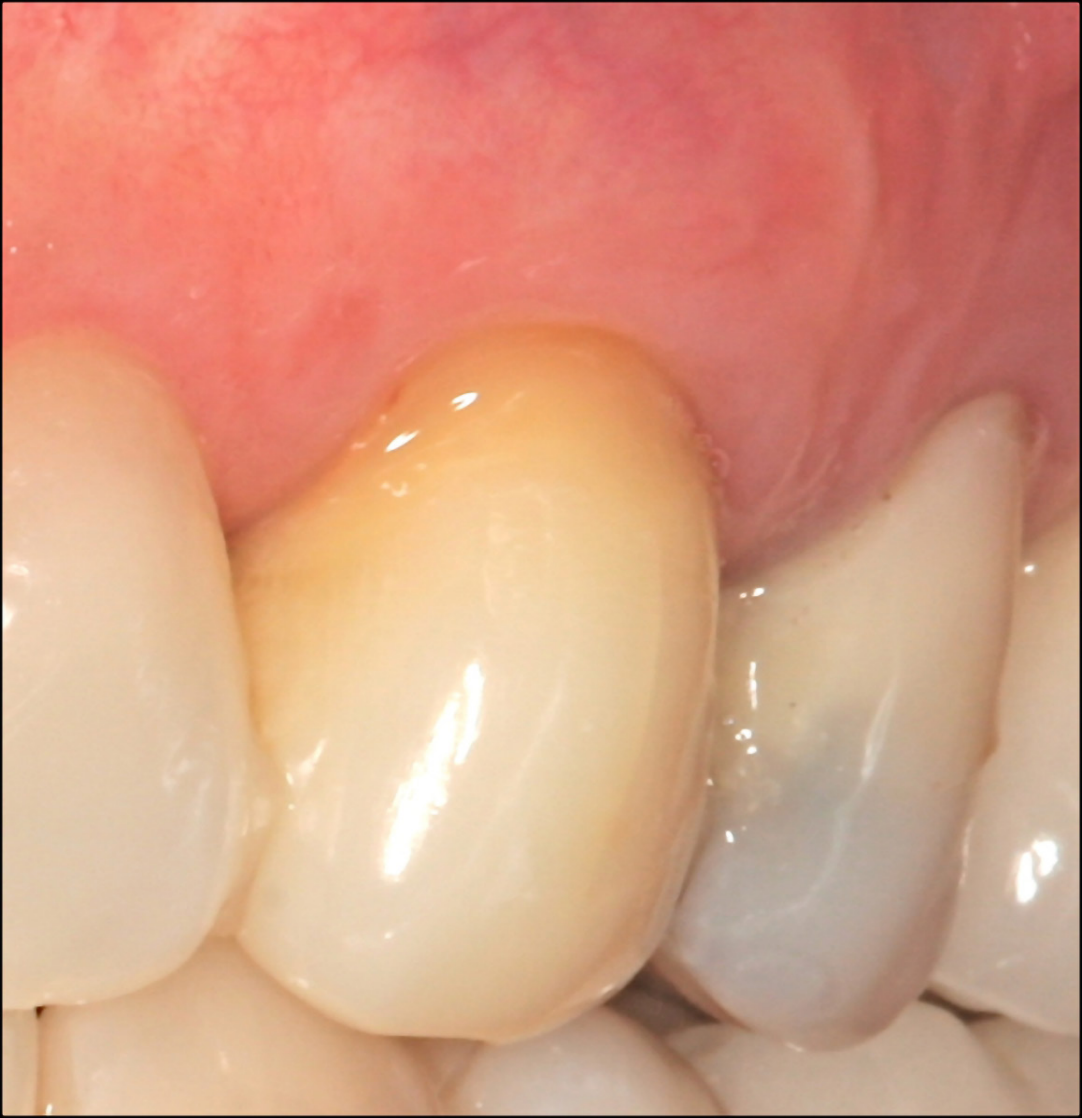
Clinical view at 18 years follow-up.

**Figure 6B:**
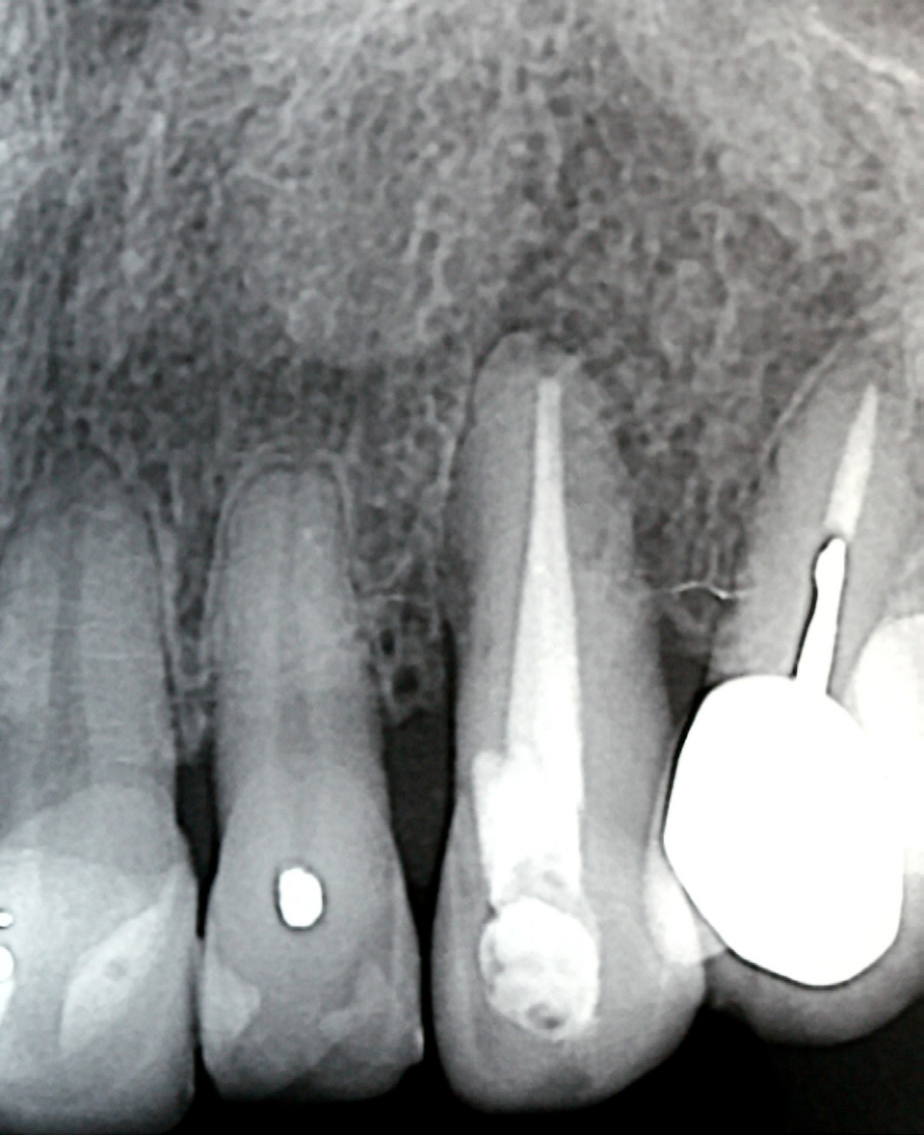
Radiograph shows a complete resolution of the lesion after 18 years.

## Discussion

In this case, the patient requested treatment for aesthetic reasons. Patients with buccal gingival recessions often complain about dental hypersensitivity and impaired aesthetics [9].

It is difficult to speculate on the mechanisms leading to GR. It can be hypothesized that the etiology of GR in this patient was related to previous orthodontic treatment with the placement of the tooth root outside the alveolar bone, causing subsequent GR. Based on observations made in an experimental study in monkeys, Wennström et al. suggested that gingiva with a thin buccolingual dimension at sites with bone dehiscence may serve as a “locus minoris resistentia” for gingival recessions development [10]. In a cohort study, Renkema et al. demonstrated a continuous increase in the number of recessions from the beginning of orthodontic treatment to five years after therapy [4]. They also found that gingival recessions were not equally distributed in the dental arches. Canines, first premolars, and first molars in the maxilla, and central incisors and first premolars in the mandible are at the highest risk for buccal gingival recessions [4].

The patient reported no changes in masticatory habits or events of hard biting. With the absence of tooth decay or fillings that could justify such a response, we propose that the orthodontic movement could cause the pulp necrosis. Most likely, the lesion started as endodontic and then reached the GR and at least partially involved the periodontal tissues. This hypothesis can be supported by Hamersky et al., who suggested that excessive and prolonged orthodontic forces, when applied to teeth, may result in loss of pulp vitality [11].

EPL can occur because the periodontium and the pulp communicate through different pathways, including dentinal tubules, lateral and accessory canals, and the apical foramen [6]. The primary endodontic lesion with secondary periodontal involvement must first be treated with endodontic therapy. This is the reason why the root canal treatment was performed immediately. Endodontic treatment has the objective to eliminate the intraradicular etiological factors and allow the healing of the periapical tissues [6]. Treatment results were evaluated after four months, and only then, the need to apply periodontal treatment was considered. Sufficient evidence in the literature reports that this treatment sequence allows sufficient time for initial tissue healing and better assessment of the periodontal condition [6].

The initial diagnosis was a primary endodontic lesion with secondary periodontal involvement. However, the degree of healing that took place following root canal therapy allowed us to rectify and determine the retrospective classification, according to Simon et al. [12], as a primary endodontic lesion. This case describes an unusual presentation of a primary endodontic lesion with wide periodontal pockets in more than one surface of the tooth and confirms that these lesions may occur in the presence of a minimal amount of calculus or plaque formation. Primary endodontic lesion heals after proper instrumentation, disinfection, and sealing of the endodontic space. Likewise, lesions of endodontic origin might have a better prognosis than those of periodontal origin [6].

Grupe and Warren introduced LPF in 1956 under the term “sliding flap” [13]. Our technique involved a partial-thickness flap and a submarginal incision at the donor site to preserve the marginal integrity of the tooth adjacent to the recession defect, and this agrees with the modifications made by Staffileno [14] and Grupe [13]. LPF is indicated for the treatment of single deep recessions associated with little or no keratinized tissue and an adequate amount of keratinized tissue lateral to the GR defect. These characteristics presented in our patient allowed us to select this technique for root coverage. In this case, the root coverage rate utilizing LPF combined with SCTG was 91% at 6 months and 79% at 2 years of follow-up and agreed with the results of previous studies [15-17]. Wennstrom included the clinical outcomes of case series studies and found the root coverage using LPF had a mean root coverage rate ranging from 41% to 74% with a mean of 68% [15]. Chambrone and Chambrone in a case series assessed the clinical results obtained with full-thickness LPF and citric acid root conditioning for the treatment of Miller Class I and II localized recession defects; the mean percentage of root coverage was 94%, and complete root coverage was 63%. However, the treatment outcome with a single deep recession may be improved by adding a connective tissue graft under the LPF [17]. Nelson demonstrated a 88% mean root coverage rate in recessions of 7 to 10 mm, during a 6- to 42-month follow-up [16].

Before surgery, complete root coverage could not be expected due to the interproximal bone loss. The presence of a graft thickness greater than 2 mm and the complete integrity of the interproximal gingiva could be considered positive prognostic factors to improve the result in the root coverage described in this case. These findings are supported by other studies [18]. The surgical technique described in this case report demonstrated that good clinical results in terms of CAL could be achieved and maintained long term.

## Conclusion

This case report illustrates how a severely compromised tooth can be successfully treated, with proper diagnosis followed by applying the correct treatment sequence. Proper diagnoses of EPL is critically important and will dictate the appropriate course of treatment.
